# Supplementary material for: Data‐independent acquisition and quantification of extracellular matrix from human lung in chronic inflammation‐associated carcinomas
Source: Proteomics. 2022 Oct 13;23(7-8):2200021. doi: 10.1002/pmic.202200021 (PMC10391693; doi:10.1002/pmic.202200021)
Supplement: Supplementary file 1 — Supporting Information [file PMIC-23-2200021-s004.pptx]

## Slide 1
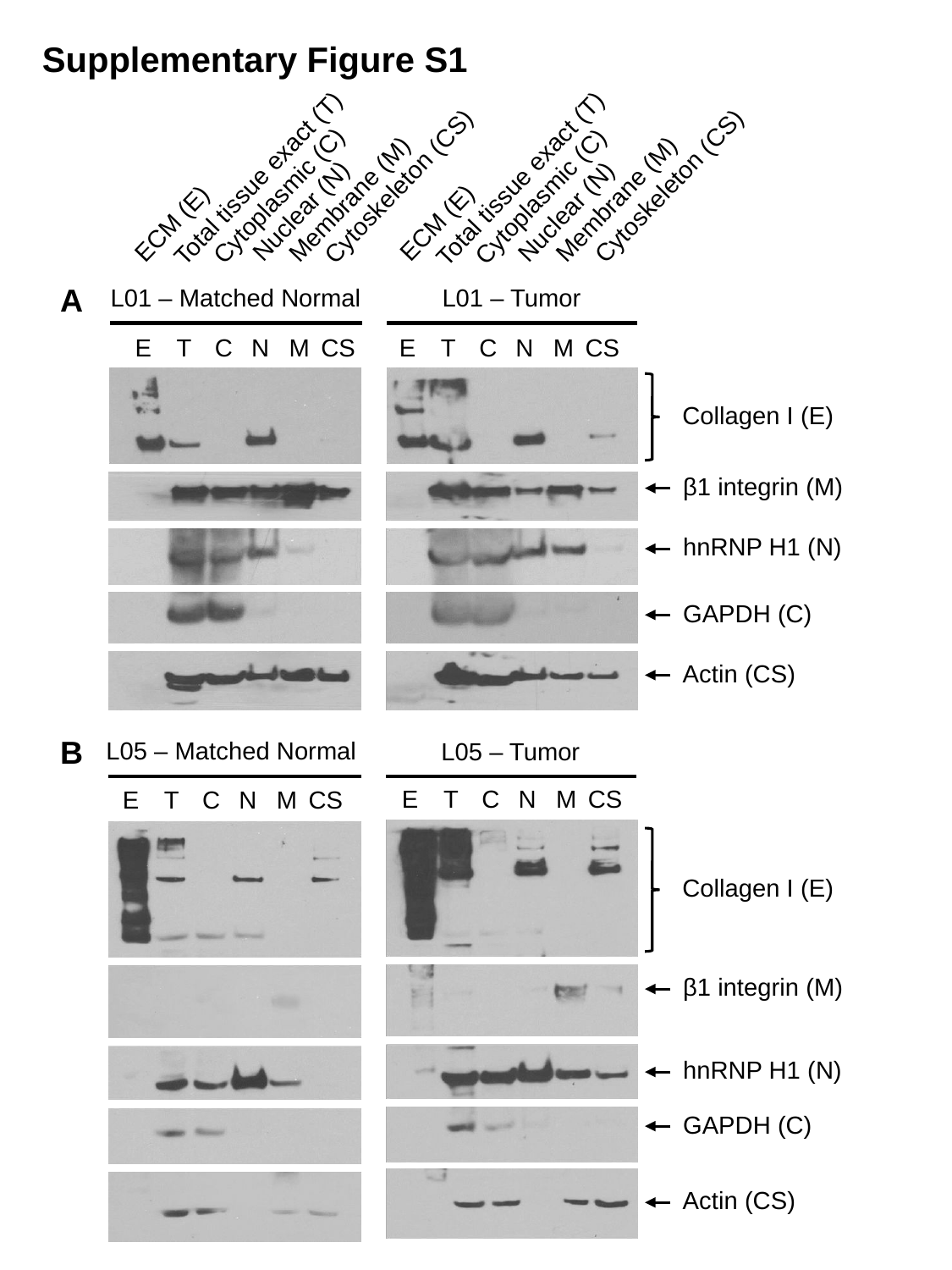

Supplementary Figure S1
Total tissue exact (T)
Total tissue exact (T)
Cytoskeleton (CS)
Cytoskeleton (CS)
Cytoplasmic (C)
Cytoplasmic (C)
Membrane (M)
Membrane (M)
Nuclear (N)
Nuclear (N)
ECM (E)
ECM (E)
A
L01 – Matched Normal
L01 – Tumor
E
T
C
N
M
CS
E
T
C
N
M
CS
Collagen I (E)
β1 integrin (M)
hnRNP H1 (N)
GAPDH (C)
Actin (CS)
B
L05 – Matched Normal
L05 – Tumor
E
T
C
N
M
CS
E
T
C
N
M
CS
Collagen I (E)
β1 integrin (M)
hnRNP H1 (N)
GAPDH (C)
Actin (CS)
