# Supplementary material for: Data‐independent acquisition and quantification of extracellular matrix from human lung in chronic inflammation‐associated carcinomas
Source: Proteomics. 2022 Oct 13;23(7-8):2200021. doi: 10.1002/pmic.202200021 (PMC10391693; doi:10.1002/pmic.202200021)
Supplement: Supplementary file 2 — Supporting Information [file PMIC-23-2200021-s002.pptx]

## Slide 1
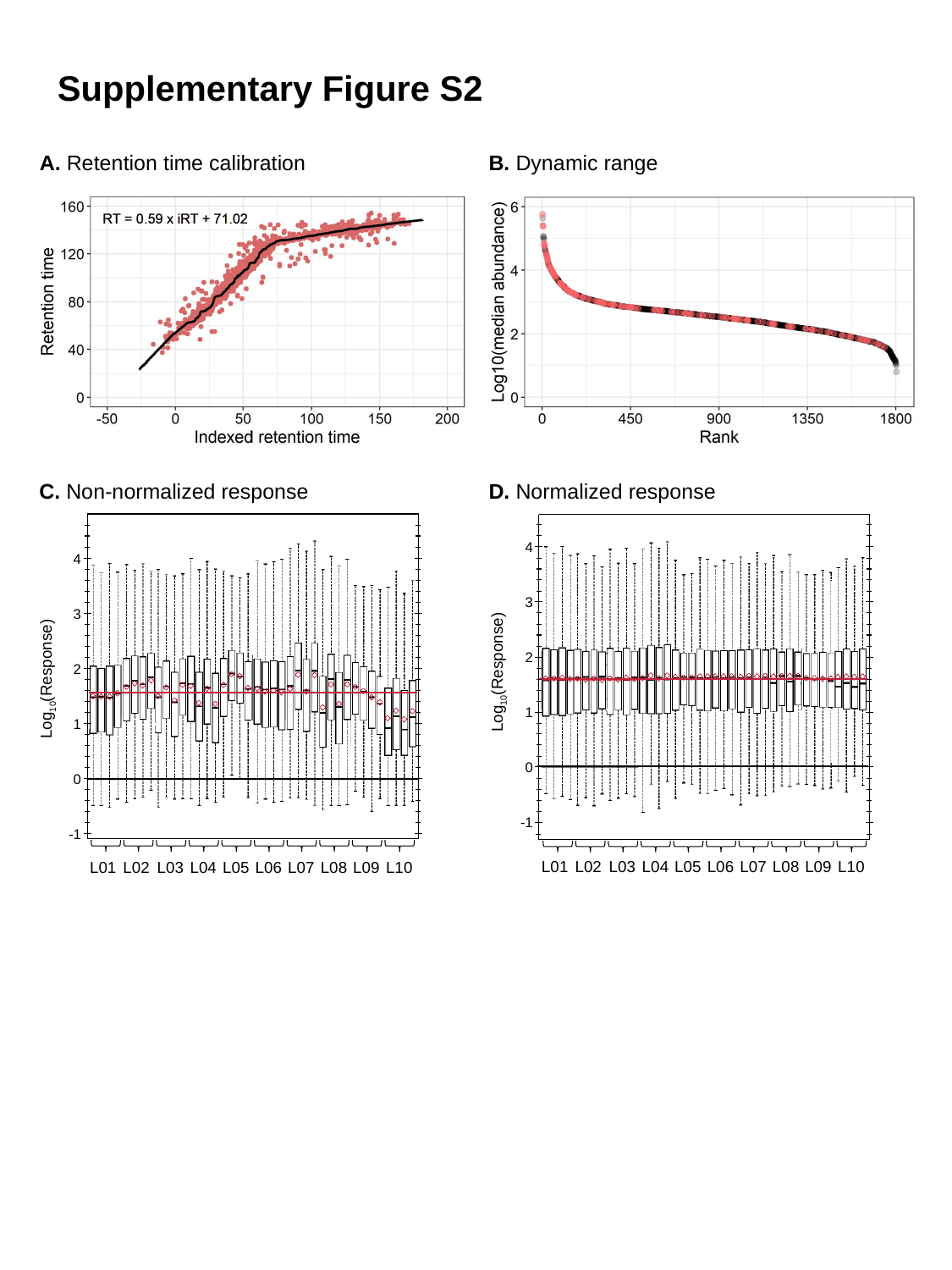

Supplementary Figure S2
A. Retention time calibration
B. Dynamic range
C. Non-normalized response
D. Normalized response
4
3
2
Log10(Response)
1
0
-1
L01
L02
L03
L04
L05
L06
L07
L08
L09
L10
4
3
2
Log10(Response)
1
0
-1
L01
L02
L03
L04
L05
L06
L07
L08
L09
L10
