# Supplementary material for: Data‐independent acquisition and quantification of extracellular matrix from human lung in chronic inflammation‐associated carcinomas
Source: Proteomics. 2022 Oct 13;23(7-8):2200021. doi: 10.1002/pmic.202200021 (PMC10391693; doi:10.1002/pmic.202200021)
Supplement: Supplementary file 3 — Supporting Information [file PMIC-23-2200021-s007.pptx]

## Slide 1
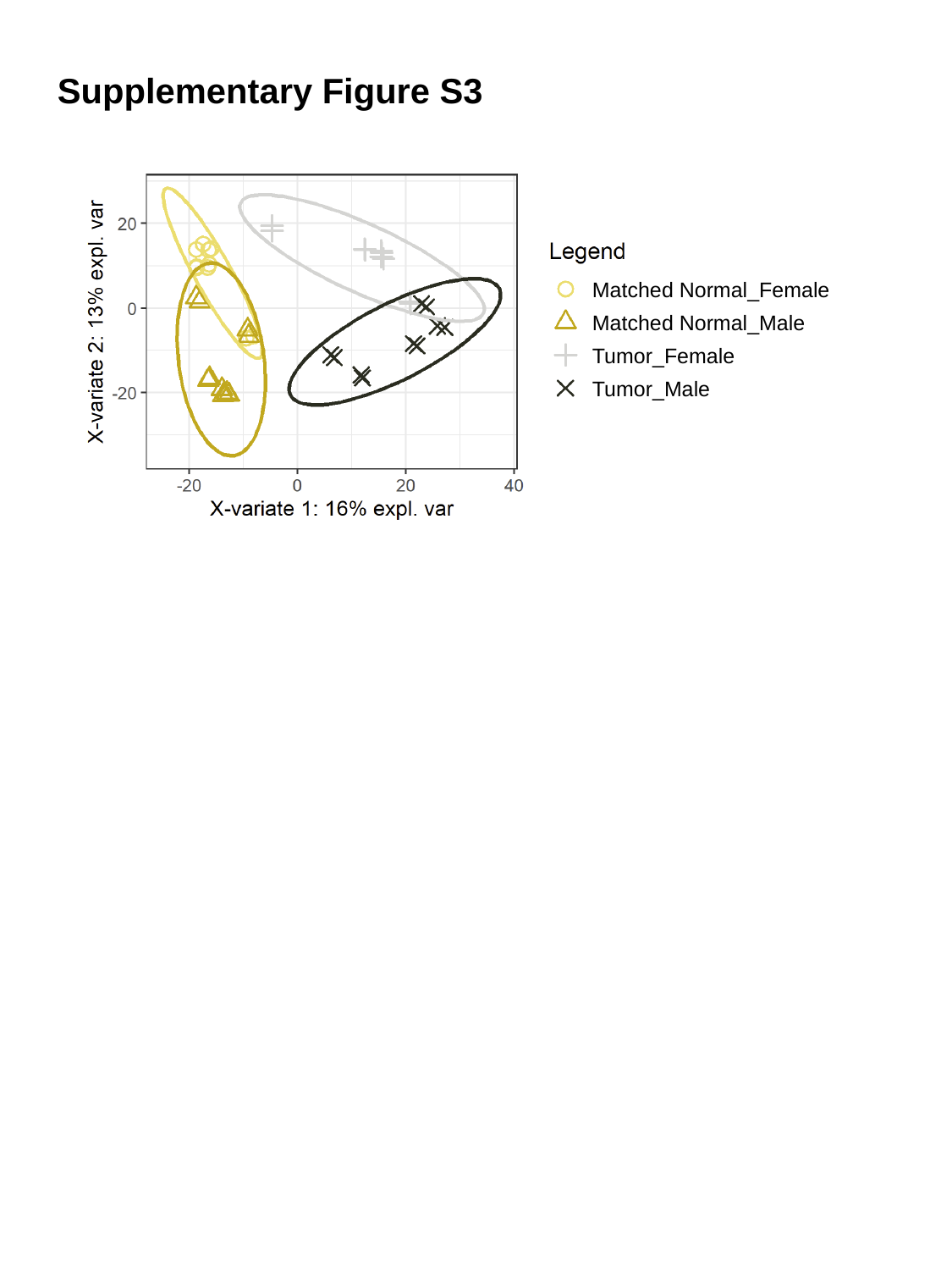

Supplementary Figure S3
Matched Normal_Female
Matched Normal_Male
Tumor_Female
Tumor_Male
