# Supplementary material for: Data‐independent acquisition and quantification of extracellular matrix from human lung in chronic inflammation‐associated carcinomas
Source: Proteomics. 2022 Oct 13;23(7-8):2200021. doi: 10.1002/pmic.202200021 (PMC10391693; doi:10.1002/pmic.202200021)
Supplement: Supplementary file 9 — Supporting Information [file PMIC-23-2200021-s008.docx]

**Data-Independent Acquisition and Quantification of Extracellular Matrix from Human Lung in Chronic Inflammation-Associated Carcinomas**

Joanna Bons^1^, Deng Pan^2^, Samah Shah^1^, Rosemary Bai^2^, Chira Chen-Tanyolac^2^, Xianhong Wang^2^, Daffolyn R. Fels Elliott^2#^, Anatoly Urisman^2^, Amy O’Broin^1^, Nathan Basisty^1^, Jacob Rose^1^, Veena Sangwan^3^, Sophie Camilleri-Broët^4^, James Tankel^3^, Philippe Gascard^2^, Lorenzo Ferri^3^, Thea D. Tlsty^2^, Birgit Schilling^1^*

**Supplementary Figure Legends**

**FIGURE S1: ECM Isolation and Quality Control.** Approximately 50 mg of tissue from the ‘Matched Normal’ and ‘Tumor’ tissues of lung squamous cell carcinomas, obtained by the Ferri team (MUHC) or the CHTN Western Division, was homogenized and the different compartment fractions were extracted based on the instruction of a compartmental protein extraction kit (Millipore, #2145). For each sample, 12 µL of total tissue extract, 24 µL of intermediate fractions, and 1/10 of ECM isolated from 50 mg of tissue were used to examine levels of representative proteins for each compartment. Images of ‘Matched Normal’ (N) and ‘Tumor’ (T) tissues from MUHC patients L01 and L05 are shown. Insoluble ECM was enriched as documented by the high levels of Collagen I in the expected ECM fractions. Representative markers for other cellular compartments (cytoplasmic: GAPDH; nuclear: hnRNP H1; membrane: β1 integrin; cytoskeleton: Actin) were barely detected in these ECM fractions supporting high purity of the isolated ECM fraction. Isolated ECM from 50 mg of each tissue was processed for further proteomic analysis.

**FIGURE S2: Quality Control and Performance of the DIA-MS Workflow.** (A) Retention time calibration obtained for a replicate of the ‘Matched Normal’ group. Pink dots correspond to peptides used for the calibration, and the black line to the non-linear calibration curve. (B) Rank plot showing the protein abundance of the 1,802 quantifiable protein groups. Abundances are depicted as median values. Pink dots correspond to matrisomal protein groups. (C-D) Boxplots of precursor abundance are displayed for each run (four runs associated with each patient) before (C) and after (D) local normalization.

**FIGURE S3: Proteomic Data Clustering - Assessment of Patient Gender.** Supervised clustering analysis using partial least squares-discriminant analysis (PLS-DA) performed on protein groups quantified in the ‘Matched Normal’ (shades of yellow) and ‘Tumor’ (shades of grey) samples collected on five female and five male patients with lung squamous cell carcinoma.

**FIGURE S4: Remodeling of the Matrisomal Proteins in Lung Squamous Cell Cancer.** List of the 66 protein groups significantly altered in ‘Tumor’ vs. ‘Matched Normal’, that are reported in the human MatrisomeDB [50].

**FIGURE S5: Heatmaps for Significantly Altered Serpins, Keratins and Desmosomal Proteins in LSCC across Individual Patients.** Heatmap showing log_2_-fold protein changes (‘Tumor’ vs. ’Matched Normal’) for each individual LSCC patient referred to as L01, L02, L03 …, and L10 assessing Core Protein Signatures. (A) SERPINB5 and (B) fifteen keratins were significantly up-regulated, while (A) SERPINB6 and (B) two keratins were significantly down-regulated in ‘Tumor’ vs. ‘Matched Normal’. In all displayed cases, Q-values (not displayed) were smaller than 2.24e-4, when comparing ‘Matched Normal’ group to ‘Tumor’ group (**Table S3**). (C) Schematic illustration of organization of keratins and desmosome proteins identified in the ECM fraction as insoluble remnants of stromal and epithelial cells.
